# Editorial: Oral Oncology: From Precise Surgery to Precision Medicine and Surgery

**DOI:** 10.3389/froh.2022.913172

**Published:** 2022-04-28

**Authors:** Zuzana Saidak, Cesare Piazza

**Affiliations:** ^1^UR7516 CHIMERE, Université de Picardie Jules Verne, Amiens, France; ^2^Centre de Biologie Humaine, Centre Hospitalier Universitaire (CHU) Amiens, Amiens, France; ^3^Department of Otorhinolaryngology, Head and Neck Surgery, University of Brescia, Brescia, Italy

**Keywords:** oral oncology, precision surgery, precise surgery, precision medicine, personalized treatment

Oral squamous cell carcinoma (OSCC) is the most common type of head and neck cancers and represents a significant medical burden worldwide (Coletta et al.). So far, surgical resection followed by appropriate reconstruction remains the first line of therapy for this kind of tumor. Precision, by definition, is the quality of being exact and accurate, and it is recognized as an essential quality of surgical care. Given the importance of achieving complete tumor resection to reach good oncological results, while preserving organ function and quality of life, oral surgery is among the medical specialties that regard precision as one of its most intrinsic qualities, as all procedures should aim to be extremely precise in their target, performance, and postoperative management. This being said, achieving surgical precision, in the sense of a safe resection with function sparing, is often challenging, especially given the variability of clinical presentations of OSCC. The main aim of this Research Topic, entitled “Oral Oncology: From Precise Surgery to Precision Medicine and Surgery”, was therefore to cover the multiple facets of surgical and medical precision, highlighting possible strategies to attain personalized, reproducible, and optimized surgical procedures. Altogether, six contributions from all over the world have been included in this special issue.

The aim was not to comprehensively list all practical solutions that are becoming increasingly available to oral surgeons, including 3D printing, cutting guides, and virtual surgical planning. Solutions for computer-assisted surgery can be used not only to achieve precise tumor resection, but also to train surgeons and prepare them for complex reconstructive procedures. Virtual surgical planning has a role to play in order to achieve more precise surgical treatment of OSCC, as shown by Crosetti et al. who herein present the results of a retrospective study examining the use and impact of virtual planning for bone resection in 20 patients with locally advanced OSCC. This study, while still preliminary, suggests that satisfactory results can be achieved in oncological terms, with a possible reduction of operative time and functional benefits. Nevertheless, virtual planning remains a difficult exercise as shown by Crosetti et al., who highlight that it is tributary to correct initial tumor staging. With this challenging complexity in mind, Alabi et al. reviewed the application of deep learning technologies in cancer detection, image classification, segmentation, and treatment planning. As the authors discuss, following the path of previous works (Paderno et al.; [1]), computer and machine learning algorithms have the potential to offer better and more individualized prognostication in view of the optimal management of OSCC.

Moving from precise surgical procedures to therapeutic stratification, two contributions examined the importance of staging in oral oncology. After many previous proposals (including from our group, as described by Piazza et al. [2]), to introduce the depth of infiltration (DOI) as an essential prognosticator in OSCC, Bresciani et al. examined the impact of the 8th TNM (8TNM) classification issued in 2017 [3], including, among others, both DOI and extranodal extension, in staging of OSCC [4]. A comparison with the former 7th TNM (7TNM) version, points to the existence of a number of differences with a valuable impact on the choice of treatment. This interesting study consolidates the conclusions of others [5, 6], by suggesting that 8TNM provides a better estimation of the risk of T3 OSCC, a T category virtually non-existent in the 7TNM. The authors, following a previous paper from Mirian et al. [7], suggest how tumor staging could be improved by closer monitoring of the number of metastatic lymph nodes, among other parameters. Another contribution, by Shetty et al. reviewed salvage surgery which, as the authors explain, remains the standard rescue treatment for recurrent, still resectable, OSCC. Shetty et al. summarize how recurrent OSCCs can be stratified and discuss new perspectives recently offered by immune checkpoint inhibitors. These two contributions highlight the essential role of accurate tumor staging for precise therapeutic stratification, key prerequisite for optimal healthcare.

Finally, the present issue addresses the importance of tumor biology within the surgical arena. The original study by Ogrinc et al. illustrates how new powerful analytical strategies, i.e., -omics, could become useful in the future surgical context. Using mass spectrometry, Ogrinc et al. provide a proof of principle that it is possible to discriminate between peritumoral and malignant tissues in tongue carcinoma. Although the study remains exploratory, Ogrinc et al. suggest the possibility of using “augmented” surgery, for example to revisit the analysis of surgical margins [8]. Finally, Galmiche et al. propose a perspective on the perioperative period, measured in days to weeks from the surgical procedure [9]. Precision medicine, i.e., individually-adapted therapy, guided by biomarkers reflecting tumor biology, is familiar to medical oncologists. An equivalent concept of precision surgery has not yet come to everyday clinical practice [10]. Galmiche et al. argue that tumor biology is likely critical in post-surgical recurrence and, conversely, that surgical/anesthetic procedures might modulate specific aspects of tumor biology during the perioperative period. Taking into account this complex interplay still requires multiple studies, but might be worthwhile given the existence of a number of already approved treatments that control inflammation, oral microbiome, as well as surgical stress during the postoperative recovery.

Overall, these valuable contributions show how a single word, “precision”, can hide a complex reality. We hope that this supplemental issue of “Frontiers in Oral Health” will open perspectives, challenge preconceived ideas, and interest the multidisciplinary community of investigators and physicians of various horizons, with the broad expertise required to achieve precise and more effective treatment of OSCC.

## Author Contributions

ZS and CP wrote the manuscript. All authors contributed to the article and approved the submitted version.

## Conflict of Interest

The authors declare that the research was conducted in the absence of any commercial or financial relationships that could be construed as a potential conflict of interest.

## Publisher's Note

All claims expressed in this article are solely those of the authors and do not necessarily represent those of their affiliated organizations, or those of the publisher, the editors and the reviewers. Any product that may be evaluated in this article, or claim that may be made by its manufacturer, is not guaranteed or endorsed by the publisher.
